# Supplementary material for: Increasing reproducibility, robustness, and generalizability of biomarker selection from meta-analysis using Bayesian methodology
Source: PLoS Comput Biol. 2022 Jun 27;18(6):e1010260. doi: 10.1371/journal.pcbi.1010260 (PMC9269905; doi:10.1371/journal.pcbi.1010260)
Supplement: Attachment — Submitted filename: reviewer_comments.docx [file pcbi.1010260.s008.docx]

The authors propose a new Bayesian framework for meta-analysis, which the authors claim to outperform the frequentist approach. The overall idea is interesting, and the real data analysis is comprehensive. I have several comments on the manuscript:

1.	In Figure 2F, why almost all samples happen to have the same expression values? Is this due to the procedure of processing the data?

2.	The authors claim that the new method is less affected by outliers. What’s the performance of the frequentist approach if the outliers are simply removed before doing the statistical tests?

3.	In Figure 4A, the cutoff of ES > 0.6 and top 10 genes seem rather arbitrary. Is there any reason why this cutoff is chosen? What’s the performance with other choices of cutoffs?

4.	In Supplemental 2, it will be interesting to see the GO enrichment analysis for genes that are only differential in Bayesian approach versus the genes that are only differential in frequentist approach, but non-differential in the other approach.

5.	Is the Bayesian model robust to different choices of prior distributions?

6.	Why there are two independent steps in the Bayesian model (ES and pooling)? Is it possible to combine them into one integrated model?
