# Supplementary material for: Increasing reproducibility, robustness, and generalizability of biomarker selection from meta-analysis using Bayesian methodology
Source: PLoS Comput Biol. 2022 Jun 27;18(6):e1010260. doi: 10.1371/journal.pcbi.1010260 (PMC9269905; doi:10.1371/journal.pcbi.1010260)
Supplement: Attachment — Submitted filename: Response to reviewers comments_V4.docx [file pcbi.1010260.s009.docx]

# **Reviewer 1**

- *In this paper, the authors describe a Bayesian meta-analysis approach that, in comparison to traditional frequentist approaches, is robust to outliers, increase the informativeness of between-study heterogeneity estimates, and reduces false positives and negatives. Overall, the paper is well-written and clearly motivated and explained. There are few comments I have that I detail section-by-section below. In summary, I think there may be room for added clarity for some figures.*

We thank the reviewer for the concise summary and the constructive feedback to improve the manuscript.

- *I commend the authors for clearly articulating this problem both from the statistical and biological perspectives.*

We thank the reviewer for the supportive comments.

- *In the last sentence of the Introduction, I suggest the authors name their method and R package.*

We thank the reviewer for the suggestion. We have included the name of the R package in the last sentence of the Introduction:

“This framework has also been developed into an R package, bayesMetaIntegrator, that is publicly available for use (<https://github.com/Khatri-Lab/bayesMetaIntegrator>).”

- *The forest plots (like in Figure 2E) are hard to read and there is limited information about them in the figure captions. For example, what do the differently sized blue boxes mean?*

Thank you for the constructive feedback. We have modified the figure to increase the font sizes to make it easier to read. We have also modified the legend for Figure 2 to include more details. For convenience, the modified legend for Figure 2 is:

“**Figure 2: Asthma meta-analysis comparison.** (A) Comparison of pooled effect sizes by the frequentist and Bayesian meta-analysis for all genes. Pearson correlation (r) = 0.94. (B) Comparison of between-dataset heterogeneity, τ^2^, by the frequentist and Bayesian meta-analysis for all genes. Pearson correlation (r) = 0.80. (C) Comparison of within-dataset effect size estimates by the frequentist and Bayesian meta-analysis for all genes. (D) Comparison between false discovery rate from the frequentist meta-analysis and probabilities from the Bayesian meta-analysis. (E) The x axes represent standardized mean difference between patients with asthma and healthy controls, computed as Hedges’ *g*, in log_2_ scale. The size of the blue rectangles is proportional to the standard error of mean difference in the study. Whiskers represent the 95% confidence interval. The orange diamonds represent overall, combined mean difference (summary effect size) for a given gene. Width of the orange diamonds represents the 95% confidence interval of overall mean difference. (F) Expression of SLC6A4 in GSE64913 and FANCI in GSE18965 in patients with asthma and healthy controls.”

- *I don’t quite see how effect sizes for FANCI are in opposite directions. The text seems to suggest the effect sizes are in opposite directions for the meta-analysis, but Figure 2E only shows different directions for one dataset.*

We apologize for the error. We meant the effect sizes are in the opposite directions for FANCI in one dataset (GSE18965) due to one outlying healthy control sample. We have modified the text as follows:

“Although both approaches estimated the effect size for *FANCI* in the same direction, the difference in statistical significance was due to the difference in estimated effect size in a single dataset (GSE18965) (**Figure 2E**).”

- *The asterisks in Figure 4 are not defined. Are these P-value adjusted for multiple comparisons?*

The p-values are not adjusted for multiple comparisons because each comparison uses different set of data and tests different hypothesis. For example, the first pair of boxplots in Figure 4B for cardiomyopathy is testing the null hypothesis that “when using **2 datasets**, genes identified using Bayesian or frequentist meta-analysis have same AUC” whereas, the second pair of boxplots is testing the null hypothesis that “when using **3 datasets**, genes identified using Bayesian or frequentist meta-analysis have same AUC.” In other words, each pair of boxplots are different hypotheses using different randomization of different datasets. We have modified the following in the legend to provide the details for asterisks in **Figure 4** as follows:

“**** p ≤ 0.0001, *** p ≤ 0.001, ** p ≤ 0.01, * p ≤ 0.05, ns: p > 0.05.”

- *How were the minimally informative prior hyperparameters elicited here? I.e., the Normal(0,3) and Uniform(0,2)?*

We thank the reviewer for this clarifying question. Reviewer 2 and 3 also have a comment in line with this comment. We analyzed several gene expression meta-analyses in our MetaSignature database (Haynes *et al.* Pacific Symposium on Biocomputing 2017; https://doi.org/10.1142/9789813207813_0015) to select the priors for the distributions of effect sizes and variance. In MetaSignature database (https://metasignature.stanford.edu), across more than 100 disease-specific meta-analyses effect sizes for genes were distributed as Normal(0, 0.6). Hence, our choice of using Normal(0,3) as a prior for effect size is minimally informative and sufficiently wide to not bias our analyses described in the manuscript. Using MetaSignature, we also found that the between-study heterogeneity, τ, was typically geometrically distributed, with most τ values closer to 0. However, after initial analyses, we found that estimates of frequentist τ and Bayesian τ were different, where frequentist τ was typically 0 for meta-analyses using datasets with small sample sizes because of overlapping confidence intervals for dataset-specific effect sizes. Therefore, our choice of Uniform(0,2) as the prior for τ is minimally informative as it is more than three times the standard deviation of effect size (i.e., 3 x 0.6 = 1.8).

Further, in response to the similar comments by Reviewer 2, who recommended a sensitivity analysis to ensure priors are non-informative, and Reviewer 3, who asked if the Bayesian model is robust to different choices of prior distributions, we performed three sensitivity analyses using the asthma datasets. For effect size, we found that, irrespective of using Normal(0, 1), Normal(0, 2) or Normal(0, 3) as the priors, the Bayesian probability remained the same. For the τ, we found that using a Uniform(0,1) had lower Bayesian probability estimates (i.e., identified more genes as significant), whereas using Uniform(0,2) or Uniform(0,3) had the same Bayesian probabilities. We have included these results as **Supplementary Figure 1** (and included here for convenience as **Figure R1**) in the revised manuscript.

# **Reviewer 2**

- *In this study, the authors propose a Bayesian methodology for gene expression meta-analysis and show strong evidence that it outperforms standard frequentist approaches, and is, in particular, much less sensitive [to] outlying data points and more sensitive to between-dataset heterogeneity. Overall, the study is well designed and the paper is well organized and written. I have no major concerns.*

We thank the reviewer for the supportive comment.

- *While I have no reservations about the final results, I think it would be helpful to include sensitivity analyses of the Bayesian prior parameters to assure that they are indeed non-informative, i.e. that the final gene rankings and posterior probabilities do not change by widening the prior further. I fully expect this to be the case.*

Please see our response to the last comment by Reviewer 1 above as this constructive suggestion by Reviewer 2 is in line with the last comment by Reviewer 1. Briefly, we performed three analyses to assure that the Bayesian prior parameters are indeed non-informative. First, across frequentist meta-analyses for >100 diseases, we found that the effect size for genes was distributed as Normal(0, 0.6), which is substantially narrower than our choice of Normal(0,3). Second, we performed sensitivity analyses as Reviewer 2 recommended. When we varied the parameters of the Normal priors for effect size, the Bayesian probabilities for differential expression remained concordant. For between-study heterogeneity, we found that using Uniform(0,1) as prior had lower Bayesian probability estimates, whereas using Uniform(0,2) or Uniform(0,3) as prior had highly concordant Bayesian probability estimates. Hence, as the reviewer predicted, the posterior probabilities and final gene rankings did not change by widening the priors further. We have included these results as **Supplementary Figure 1** (included here for convenience as **Figure R1**) in the revised manuscript.

**Figure R1 (Supplementary Figure 1 in revised manuscript). Sensitivity analysis of Bayesian priors for effect size (ES) and heterogeneity (tau).** Distribution of **(A)** summary effect size and **(B)** tau (measured as standard deviation of summary effect size) using different number of datasets for meta-analysis. **(C-D)** Comparison of Bayesian probability for differential expression of a gene using different Normal priors for summary effect size. **(E-F)** Comparison of Bayesian probability for differential expression of a gene using different Uniform priors for heterogeneity.

We have also included the following text in the manuscript describing the sensitivity analysis:

“We chose priors for effect size and between-study heterogeneity using 122 previous gene expression meta-analyses (**Figure S1A-B**)^46^. Sensitivity analysis of the priors found that when we varied the parameters of the Normal priors for effect size, the Bayesian probabilities for differential expression remained concordant (**Figure S1C-D**). For between-study heterogeneity, we found that using Uniform(0,1) as prior had lower Bayesian probability estimates (**Fig. S1E**), whereas using Uniform(0,2) or Uniform(0,3) as prior had highly concordant Bayesian probability estimates (**Fig. S1F**). Hence, the final gene rankings and posterior probabilities did not change by widening the priors further.”

**Reviewer 3**

- *The authors propose a new Bayesian framework for meta-analysis, which the authors claim to outperform the frequentist approach. The overall idea is interesting, and the real data analysis is comprehensive. I have several comments on the manuscript:*

We thank the reviewer for supportive comments.

- *In Figure 2F, why almost all samples happen to have the same expression values? Is this due to the procedure of processing the data?*

We believe that almost all samples have same low expression values due to both technical and biological reasons. As the reviewer is likely aware, when gene expression values quantified using microarray, they are mean florescence intensity (MFI) values. The Y-axis in **Figure 2F** clearly shows that these are MFI values corresponding to low expression values. These very low values are presented as very low intensity (MFI) values. Hence, very low values for most samples suggest that it is highly likely that the gene is generally not expressed in these samples (bronchial epithelial cells), which leads to constant values except for a very small number of samples.

- *The authors claim that the new method is less affected by outliers. What’s the performance of the frequentist approach if the outliers are simply removed before doing the statistical tests?*

We respectfully argue against removing the outliers before doing the statistical tests. First, as we mentioned in the Introduction section of our manuscript, “*there are three sources of heterogeneity in the real-world patient population: biological (age, sex, tissue, cell type), clinical (treatment, disease duration, comorbidities), and technical (experimental protocol, batch effects).*” Given the breadth of biological and clinical heterogeneity in the real-world population, when integrating multiple independent data sets, a few samples, whether healthy or with a disease, across datasets are expected to have slightly different expression. It is impossible to know whether the outlying value observed for a gene in a subset of samples in one group is due to biological, clinical, or technical heterogeneity. In fact, an outlier may not be an outlier (e.g., Figure 2F), but a representative of the variability observed in the real-world population in a small number of subjects.

**Figure R2**. **Principal component analysis showed that GSM1583251 is not an outlier sample although it had outlier expression of SLC6A4 compared to other samples in the dataset.** GSM1583251 is highlighted in red.

Second, most of the time, as was the case here, it is only a subset of genes that a sample has different values, not for all genes. For instance, the sample, GSM1583251, which was an outlier for *SLC6A4,* was not an outlier for 18,053 other genes. Principal component analysis showed that the first two principal components only explained 25.7% variance in this dataset and GSM1583251 was not an outlier (**Figure R2**). Hence, if we were to remove outlier samples as the reviewer suggested, we will remove different sets of samples for different genes, which must not be done. Given that the outliers very likely represent the real-world biological and clinical heterogeneity and removing different set of samples for different genes is not appropriate, we have refrained from the analysis suggested by the reviewer.

- *In Figure 4A, the cutoff of ES > 0.6 and top 10 genes seem rather arbitrary. Is there any reason why this cutoff is chosen? What’s the performance with other choices of cutoffs?*

This is a point is well taken. In response to the reviewer’s comment, we performed two analyses using the TB and cardiomyopathy datasets to investigate whether varying the effect size threshold or the number of genes used for building a classifier would affect our conclusions. We analyzed 4 datasets for both diseases using frequentist and Bayesian approach. Next, we selected differentially expressed genes as before but by varying the absolute effect size from 0.4 to 1.1. We found that the genes identified as significant by Bayesian meta-analysis had significantly higher AUROC compared to those identified by a frequentist meta-analysis. When we varied the number of genes from 10 to 200, genes identified using the Bayesian meta-analysis approach had consistently higher AUROCs than the genes identified using the frequentist approach. Overall, both analyses showed that irrespective of the effect size threshold or the number of genes used for building a classifier, Bayesian approach identified genes that consistently had higher accuracy. We have included these results as **Supplementary Figure 2** (included here for convenience as **Figure R3**) in the revised manuscript. We have also included the following text in the manuscript:

“When we varied the effect size threshold (0.4 to 1.1) and the number of selected genes (10 to 200), while keeping the number of datasets used for analysis constant at 4, the genes selected using the Bayesian approach consistently led to higher accuracy (**Fig S2**).”

**Figure R3 (Supplementary Figure 2 in revised manuscript)**. Sensitivity analysis of effect size threshold and a different number of genes on classifier AUROC.

- *In Supplemental 2, it will be interesting to see the GO enrichment analysis for genes that are only differential in Bayesian approach versus the genes that are only differential in frequentist approach, but non-differential in the other approach.*

In response to the reviewer’s comment, we have added this analysis to **Supplementary Figure 4**. Using the same thresholds as before, we did not find any large substantial differences in the pathways identified by using the genes identified in only one of the methods but not the other.

- *Is the Bayesian model robust to different choices of prior distributions?*

Please see our response to the last comment by Reviewer 1 and Figure R1. As we demonstrated above, the Bayesian model is robust to different choices of prior distributions.

- *Why there are two independent steps in the Bayesian model (ES and pooling)? Is it possible to combine them into one integrated model?*

We thank the reviewer for the clarifying comment in a separate email that “*if it would be possible to design one integrated model that simultaneously accounts for both dataset-specific and summary-level effect size? For example, the data generating mechanism could be a hierarchical model where the summary-level effect size generates the dataset-specific effect size, and the dataset-specific effect size further generates the observed data. The summary-level effect size is the quantity of interest and the dataset-specific effect size could be treated as latent variables.*”

We apologize for the confusion and not explicitly stating that it is exactly what we have done in our manuscript Specifically, we are using a hierarchical model in the pooling step. We create a pooled summary distribution using Gibbs sampling from the individual dataset-specific distributions. During this process, we also treat the dataset-specific distributions as a latent variable – regenerating the dataset-specific distributions from the summary distribution – allowing the dataset-specific distributions to shift depending on the estimate of the pooled summary distribution and between-study heterogeneity. We have modified the manuscript to explain this more clearly:

“Using the hierarchical model structure, once the pooled distribution is estimated, we adjusted the individual dataset effect size distributions based on the summary distribution, akin to a random effects model in frequentist meta-analysis.”

***Have the authors made all data and (if applicable) computational code underlying the findings in their manuscript fully available?***

*Reviewer #1: Yes*

*Reviewer #2: Yes*

*Reviewer #3:* ***No:*** *Authors provided a Github repo for the software (*[*https://github.com/Khatri-Lab/bayesMetaIntegrator*](https://github.com/Khatri-Lab/bayesMetaIntegrator)*), but the codes and data to reproduce the analyses in this manuscript have not been provided.*

We have added code to create these analyses to the GitHub, in addition to the software. All datasets used in our analyses, including gene expression, are publicly available from the NCBI Gene Expression Omnibus (GEO). We have provided the accession ID for each dataset in **Supplementary Table 1**.
